# Aging mitigates the severity of obesity-associated metabolic sequelae in a gender independent manner

**DOI:** 10.1038/s41387-021-00157-0

**Published:** 2021-06-07

**Authors:** Maria E. Moreno-Fernandez, Vishakha Sharma, Traci E. Stankiewicz, Jarren R. Oates, Jessica R. Doll, Michelle S. M. A. Damen, Maha A. T. A. Almanan, Claire A. Chougnet, David A. Hildeman, Senad Divanovic

**Affiliations:** 1 Department of Pediatrics, University of Cincinnati College of Medicine, Cincinnati, OH 45220 USA; 2 Division of Immunobiology Cincinnati Children’s Hospital Medical Center, Cincinnati, OH 45229 USA; 3 Immunology Graduate Program Cincinnati Children’s Hospital Medical Center and the University of Cincinnati College of Medicine, Cincinnati, OH 45220 USA; 4 Center for Transplant Immunology, and Inflammation and Tolerance, Cincinnati Children’s Hospital Medical Center Cincinnati, Ohio, 45229 USA; 5 Center for Inflammation and Tolerance, Cincinnati Children’s Hospital Medical Center, Cincinnati, Ohio, 45229 USA

**Keywords:** Obesity, Diabetes, Immunology

## Abstract

**Background:**

Understanding gender-associated bias in aging and obesity-driven metabolic derangements has been hindered by the inability to model severe obesity in female mice.

**Methods:**

Here, using chow- or high fat diet (HFD)-feeding regimens at standard (T_S_) and thermoneutral (T_N_) housing temperatures, the latter to model obesity in female mice, we examined the impact of gender and aging on obesity-associated metabolic derangements and immune responsiveness. Analysis included quantification of: (i) weight gain and adiposity; (ii) the development and severity of glucose dysmetabolism and non-alcoholic fatty liver disease (NAFLD); and (iii) induction of inflammatory pathways related to metabolic dysfunction.

**Results:**

We show that under chow diet feeding regimen, aging was accompanied by increased body weight and white adipose tissue (WAT) expansion in a gender independent manner. HFD feeding regimen in aged, compared to young, male mice at T_S_, resulted in attenuated glucose dysmetabolism and hepatic steatosis. However, under T_S_ housing conditions only aged, but not young, HFD fed female mice developed obesity. At T_N_ however, both young and aged HFD fed female mice developed severe obesity. Independent of gender or housing conditions, aging attenuated the severity of metabolic derangements in HFD-fed obese mice. Tempered severity of metabolic derangements in aged mice was associated with increased splenic frequency of regulatory T (T_reg_) cells, Type I regulatory (Tr1)-like cells and circulating IL-10 levels and decreased vigor of HFD-driven induction of inflammatory pathways in adipose and liver tissues.

**Conclusion:**

Our findings suggest that aging-associated altered immunological profile and inflammatory vigor may play a dominant role in the attenuation of obesogenic diet-driven metabolic dysfunction.

## Introduction

Aging, a key risk factor for development of numerous chronic diseases, is linked with weight gain/obesity, redistribution of white adipose tissue (WAT) towards abdominal fat and functional and structural deterioration of vital organs^1^. Both aging and obesity contribute to the development of various metabolic derangements including type II diabetes (T2D), cardiovascular disease (CVD) and non-alcoholic fatty liver disease (NAFLD)^2–4^. Gender also differentially impacts obesity and pathophysiology of metabolic derangements^5^. Women, compared to men, are at higher risk for becoming obese across their life span^6^. WAT distribution varies between men and women, with males and post-menopausal women exhibiting increased amounts of visceral fat, while pre-menopausal women exhibit increased amounts of subcutaneous fat^7^. Further, pre-menopausal females display milder metabolic disease compared to males^8–10^.

The inability to model robust obesity in C57BL/6 wild type (WT) female mice limits the interrogation of the influence of gender in obesity-driven metabolic derangements^11–13^. Such difficulties can be attributed to the housing temperatures ubiquitously employed in mouse husbandry (20–23 °C, standard housing temperature T_S_)—a temperature range that dramatically alters mouse physiology which dampens animal weight gain and their development of metabolic sequelae. Importantly, housing mice at their thermoneutral zone (28–33 °C, T_N_) allows for modeling of severe obesity in C57BL/6 WT female mice and the investigation of metabolic disease pathogenesis^13,14^.

Aging and obesity are associated with an altered immunological environment. The obesity skewed immune responsiveness, which broadly favors proinflammatory cytokine (e.g., IL-6, TNF) and chemokine (e.g., CCL2) production, is directly linked with obesity-driven metabolic derangements^15,16^. Aging increases proinflammatory cytokine (e.g., IL-6, IFNγ) levels^17^ and is associated with a decrease in naive T and B cell pools, natural killer (NK) cell cytotoxicity, and innate immune cell function^18^. Additionally, aging promotes accumulation of peripheral and WAT regulatory T cells that express high levels of CD25 (T_regs_) and increased IL-10 production^19^— immune mediators associated with healthy aging, WAT homeostasis, and insulin-sensitizing effects^20,21^. Thus, given the clinical and public health significance of obesity, the ever-increasing numbers of elderly people worldwide, and the therapeutic promise for targeting pathogenic immune responses, better insights into the underlying basis of combined aging and gender-dependent effects on obesity-driven metabolic derangements are needed.

In this study we aimed to examine the combinatory role of aging and gender-bias on weight gain, development of metabolic derangements, and altered immune responsiveness. The utility of T_N_ housing allowed for assessment of these variables in obese young and aged female mice. We show that aging, in a gender independent manner, was accompanied by increases in body weight, and WAT expansion and inflammation. Under HFD feeding regimen, T_N_ housing was required to induce robust obesity in young female mice and allowed for comparison of metabolic derangement severity across gender and aging. Employing such experimental permutations, we show that, independent of gender or housing conditions, the severity of metabolic derangements was attenuated in aged mice compared to young counterparts. Tempered severity of metabolic derangements correlated with increased splenic frequency of regulatory T (T_reg_) cells, type I regulatory (Tr1)-like cells and circulating IL-10 levels, and decreased vigor of HFD-driven induction of inflammatory pathways in adipose and liver tissues.

## Material and methods

### Mice

All male and female mice were on a C57BL/6 background. Young mice (2 months) were bred in house. Middle-aged mice (14 months) mice were obtained from the National Institute on Aging (NIA) colony located at Charles River Laboratories (Wilmington, MA, USA). All studies were approved by the Cincinnati Children’s Hospital Medical Center (CCHMC) Institutional Animal Care and Use Committee (IACUC). Animal care was provided in accordance with the Guide for the Care and Use of Laboratory Animals. Mice were housed either at 22 °C or 30 °C for the duration of experiments as indicated.

### Obesity models

All diet-induced obesity (DIO) studies were performed as previously reported^13,22–25^ with animals fasted overnight prior to terminal harvests. Briefly, 2- and 14-months old male and female mice were fed either an irradiated high-fat diet (HFD; Research Diets #D12492; 60% of calories from fat) or a chow diet (Chow; LAB Diet #5010; 13% calories from fat) ad libitum and had free access to water for up to 20 weeks. At the time of analysis, the animals were approximately 7 months and 19 months old respectively. Thus, young adult mice were 3–7 months of age and old mice were 18–24 months of age. For all studies, body weight and food consumption were quantified weekly. For T_N_ (30 °C) studies, mice were acclimated for 2 weeks before initiation of HFD feeding regimen.

### Glucose metabolism phenotyping

All mice were fasted overnight before completion of fasting glucose, glucose tolerance, and insulin tolerance measurements. Glucose and insulin tolerance tests were done as previously described^13,22,24^. Briefly, glucose tolerance test was determined by intraperitoneal (i.p.) injections of 10 μl of a 10% dextrose solution per gram of bodyweight and insulin tolerance test was determined by i.p. injections of 10 μl of 0.15 U/ml solution of insulin (Novolin) per gram of body weight. Blood glucose levels were measured after 0, 20, 40, 60, 90 and 120 min after injection.

### Hepatic function and phenotyping

Hepatic triglycerides were quantified using Triglyceride Reagent and Triglyceride Standards (Pointe Scientific) as previously described^13,22–24,26^. Serum alanine transaminase (ALT) levels were quantified using ALT Reagent and Catatrol I and II (Catachem). For histology, liver tissue was fixed in 10% buffered formalin, and stained with H&E^13,22,23,26,27^.

### T_reg_ depletion

Mice fed HFD, were treated intraperitoneally (i.p.) every other day for 2 weeks, with 100 μg/mouse of anti-mouse CD25 mAb (Clone PC-61.5.3 BioXCell). Glucose tolerance test was performed before and after the treatment as described above.

### Neutralization of IL-10 and IL-10R

Mice fed HFD, were treated intraperitoneally (i.p.) every other day for 2 weeks, with 100 μg/mouse of anti-mouse IL-10 mAb (clone PC-61.5.3, BioXCell) or with 500 μg/mouse of anti-mouse IL-10R (clone 1B1.3A, BioXCell) mAb^19^. Glucose tolerance test was performed before and after treatment as described above.

### Quantitative Reverse-Transcription Polymerase Chain Reaction (qRT-PCR)

mRNA expression of genes was determined as previously reported^13,22–25^. Briefly, adipose and liver tissue samples were homogenized in TRIzol (Invitrogen), RNA was extracted, reverse transcribed to complementary DNA (Verso cDNA Synthesis Kit, Thermo Scientific), and subjected to qPCR analysis (Light Cycler 480 II; Roche). The following primer pairs were used (Life Technologies): *CCL2* For AGATGCAGTTAACGCCCCAC and Rev TGTCTGGACCCATTCCTTCTTG; *CCL22* For TGGAGTAGCTTCTTCACCCA and Rev TCTGGACCTCAAAATCCTGC; *F4/80* For CTTTGGCTATGGGCTTCCAGTC and Rev GCAAGGAGGACAGAGTTTATCGTG; *Il6* For TGGTACTCCAGAAGACCAGAGG and Rev AACGATGATGCACTTGCAGA; *Tnf-alpha* For CCAGACCCTCACACTCAGATCA and Rev CACTTGGTGGTTTGCTACGAC; *p22phox* For CCTGCSGCGATAGAGTAGGC and Rev TCATGGGGCAGATCGAGT. mRNA expression of each gene was compared to β–actin expression: For GGCCCAGAGCAAGAGAGGTA and Rev GGTTGGCCTTAGGTTTCAGG (an endogenous housekeeping gene control).

### Flow cytometry

Immune infiltration into adipose and liver tissues and splenic composition was quantified by flow cytometry as previously described^13,22–25^. Briefly, single cell suspensions from indicated tissues were obtained by enzymatic digestion. To determine cytokine production, total single cells were stimulated for 5 h with 50 ng/ml PMA (Sigma-Aldrich, St. Louis, MO) and 1 μg/ml Ionomycin (Calbiochem), in presence of brefeldin A (10 μg/mL, Sigma-Aldrich). Subsequently, flow cytometry was used to enumerate immune cell populations. Briefly, cells were incubated in PBS supplemented with 2% FBS and were stained with Live/Dead stain (Zombie UV Dye: Biolegend) and with directly conjugated monoclonal antibodies to CD3-AF700(145-2C11), TCRβ-BV711 (H57-597), CD8-PECʏ−7 (53-6.7), CD4-APC (RM4-5) (all antibodies from eBioscience) for 30 min. For intracellular staining, cells were fixed and permeabilized using eBioscience buffer and stained with FoxP3-PB (FJK-16s) and IL-10-PE (JES5-16E3). Flow cytometry data were collected using an LSR Fortessa (BD) flow cytometer and analyzed using FlowJo X software (vX0.7) and FACS Diva Software.

### Statistical analysis

Statistical tests were employed for all data sets with similar variance. For normally distributed data, Student’s *t* test was used when comparing two groups. One-way ANOVA was used for three or more groups. Statistical analysis was completed using Prism 9 (GraphPad Software, Inc.). All values are represented as means ± standard error mean (SEM). No power analysis was performed to determine the sample size. The sample size in each study was based on experience with previous studies employing dietary challenges in mice. No animals were excluded from the analyses and none of the studies were blinded.

## Results

### Nutritional excess uncovers aging-dependent skewing of metabolic disease severity in male mice

**Fig. 1:**
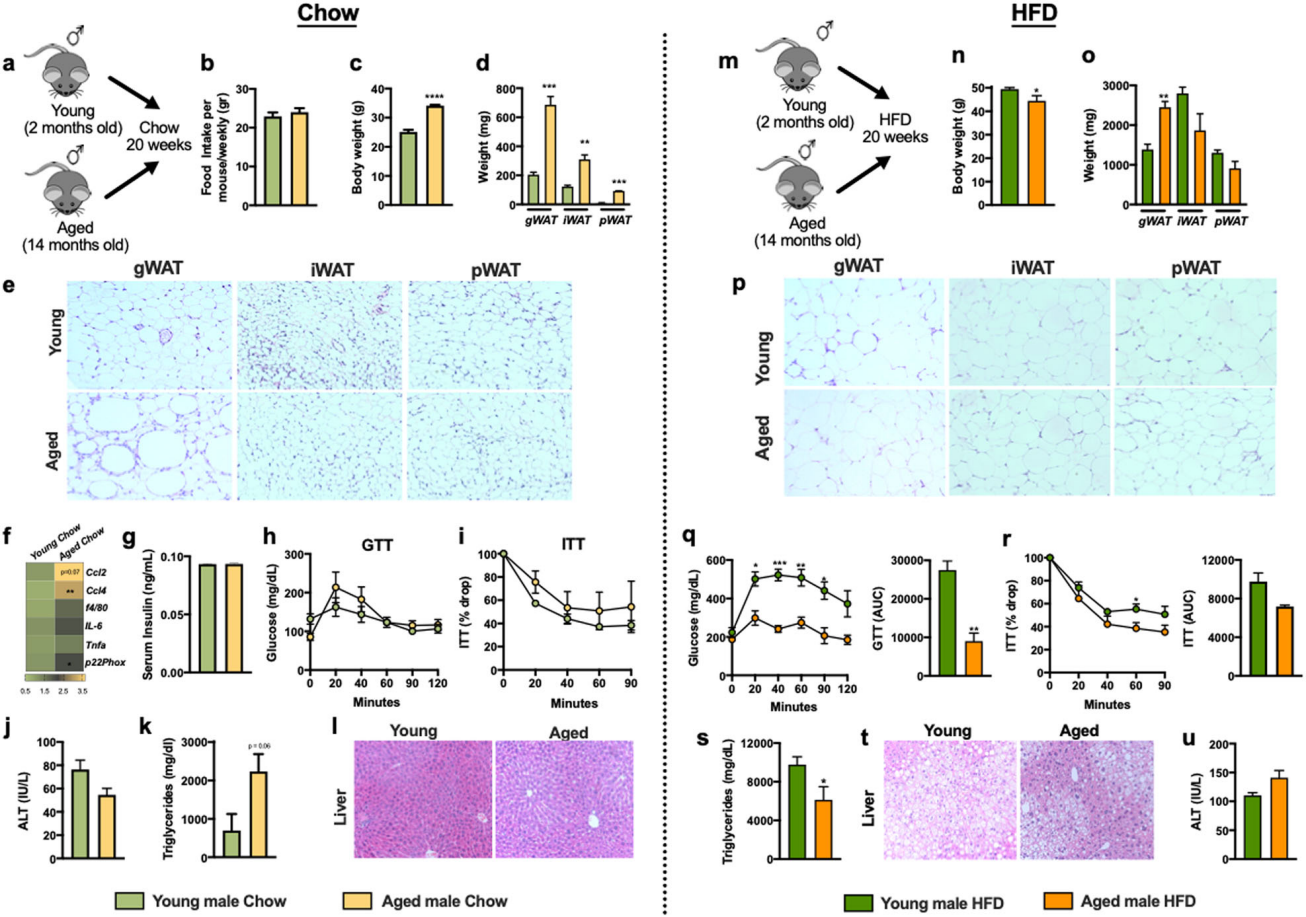
Aging mitigates metabolic disease severity in male mice. 2-month-old (Young) and 14-month-old (Aged) male C57BL/6 mice, (*n* = 4–5/condition) were housed at 22 °C (T_S_) and fed a Chow or a HFD for up to 20 weeks. **a** Schematic representation of aged model used. **b** Weekly food intake. **c** Body weight at time of harvest. **d** White adipose tissue (WAT) weight: gonadal WAT (gWAT), inguinal WAT (iWAT) and perirenal WAT (pWAT). **e** gWAT, iWAT and pWAT hematoxylin eosin (H&E) staining. **f** Heatmap of gWAT mRNA levels of *Ccl2, Ccl4*, *f4/80*, *Il6, Tnf and p22phox* (*n* = 3) fold change relative to chow fed young male. **g** Fasting serum insulin levels. **h** Glucose tolerance test (GTT). **i** Insulin tolerance test (ITT). **j** Liver triglycerides. **k** Liver H&E staining. **l** Serum Alanine transaminase (ALT) levels. **m** Schematic representation of DIO model used. **n** Body weight at time of harvest. **o** White adipose tissue (WAT) weight: gonadal WAT (gWAT), inguinal WAT (iWAT) and perirenal WAT (pWAT). **p** gWAT, iWAT and pWAT hematoxylin eosin (H&E) staining. **q** Left, glucose tolerance test (GTT) and Right, GTT area under the curve (AUC). **r** Left, insulin tolerance test (ITT) and Right, ITT area under the curve (AUC). **s** Liver triglycerides. **t** Liver H&E staining. **u** Serum Alanine transaminase (ALT) levels. A representative of 2 individual experiments. Data represent means + SE. **b**, **c**, **e–h**, **j**, **n**, **o**, **q–s**, **u**) Unpaired Student *t* test *P < 0.05, **P < 0.005, ***P < 0.0005 and ****P < 0.0001.

Aging is associated with an increase in abdominal obesity and the development of metabolic derangements^28–30^. To examine the impact of aging on induction of metabolic derangements, young (7 months at time of analysis) and aged (19 months at time of analysis) male mice were housed a standard temperature (T_S_; 22 °C) (Fig. 1a). Despite similar food intake (Fig. 1b), aged mice fed a chow diet (CD) exhibited increased total body weight (Fig. 1c) and expansion of gonadal white adipose tissue (gWAT), inguinal WAT (iWAT), and perirenal WAT (pWAT) weight (Fig. 1d, e). Aged male mice also exhibited increase in AT mRNA expression of key macrophage recruiting chemokines (e.g., *Ccl2, Ccl4*), reactive oxygen species (ROS) production (e.g., *p22phox)* and modest increase in markers of macrophage accrual (e.g., *f4/80)* and inflammation (e.g., *Il6 and Tnf*) (Fig. 1f). Serum insulin levels (Fig. 1g), responsiveness to exogenous glucose and insulin challenge (Fig. 1h, i), and liver function (Fig. 1j) were not altered despite mild increase in liver triglycerides (Fig. 1k, l). Together these data suggest that under CD feeding regimen, despite WAT expansion and mild tissue inflammation, aging alone is not sufficient to induce development of metabolic diseases.

Given the association between aging, weight gain, and chronic inflammation, we hypothesized that aging coupled to nutritional excess would worsen metabolic derangements in mice. To test this hypothesis, young (2 months) and aged (14 months) male mice were maintained at standard housing temperature (T_S_; 22 °C) and fed a high fat diet (HFD) for 20 weeks (young mice −7 months at time of analysis and aged mice-19 months at time of analysis. Figure 1m). Despite robust weight gain in both young and aged male mice (Fig. 1n) and similar food intake (Supplementary Fig. 1a), aging impeded total weight gain (Supplementary Fig. 1b). HFD fed aged male mice exhibited dichotomous WAT distribution with significantly expanded gWAT but similar iWAT and pWAT (Fig. 1o) which correlated with the adipocyte size (Fig. 1p). Aged male mice, compared to young counterparts, had improved glucose handling and tolerance (Fig. 1q, r) and lower fasting insulin levels (Supplementary Fig. 1c). Aged male mice also exhibited modest decreases in mRNA expression of inflammatory mediators (e.g., *Ccl4, Ccl2*, *f4/80, Il6, Tnf* and *p22phox*) in the WAT compared to young counterparts (Supplementary Fig. 1d). Importantly, induction of WAT inflammation was mitigated by aging as the ratio of the vigor of inflammatory mediator expression (e.g., *Ccl4, Ccl2*, *f4/80, Il6, Tnf* and *p22phox*) from chow to HFD fed mice was significantly reduced in aged mice compared to young counterparts (Supplementary Fig. 1e). Moreover, aged male mice fed HFD had decreased hepatic steatosis (Fig. 1s, t) but similar hepatocellular damage (Fig. 1u) compared to young male mice. Congruently, HFD fed aged and young male mice exhibited similar expression of inflammatory mediators (e.g., *Ccl4, Ccl2*, *f4/80, Il6, Tnf* and *p22phox*) in the liver (Supplementary Fig. 1f). Together, these data suggest that aging in male mice restricts HFD-driven weight gain and mitigates glucose intolerance, hepatic steatosis and tissue inflammation in obesity.

**Fig. 2:**
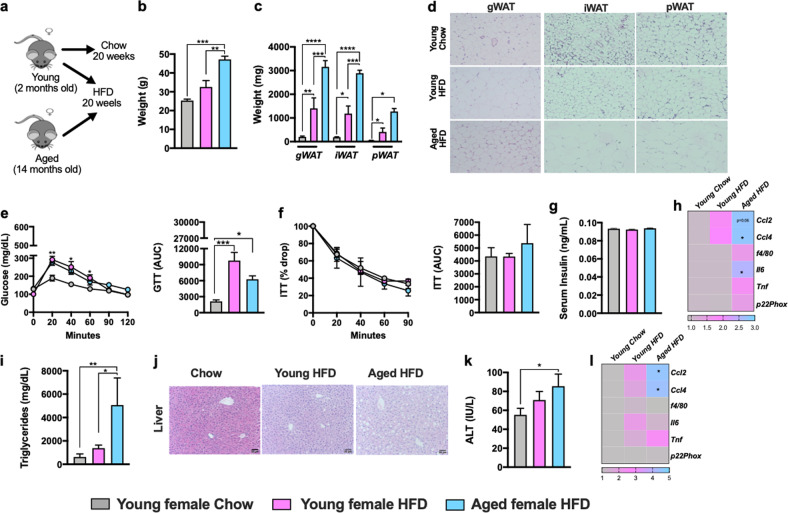
HFD amplifies aging-associated weight gain and uncovers aging-dependent skewing of metabolic disease severity in female mice. 2-month-old (Young) and 14-month-old (Aged) female C57BL/6 mice, (*n* = 3–5/condition) were housed at 22 °C (T_S_) and fed Chow or HFD for up to 20 weeks. **a** Schematic representation of DIO model used. **b** Body weight at time of harvest. **c** White adipose tissue (WAT) weight: gonadal WAT (gWAT), inguinal WAT (iWAT) and perirenal WAT (pWAT). **d** gWAT, iWAT and pWAT hematoxylin eosin (H&E) staining. **e** Left, glucose tolerance test (GTT) and Right, GTT area under the curve (AUC). **f** Left, insulin tolerance test (ITT) and Right, ITT area under the curve (AUC). **g** Serum insulin levels. **h** Liver triglycerides. **i** Liver H&E staining. **j** Serum Alanine transaminase (ALT) levels. **k** Heatmap of WAT mRNA levels of *Ccl2, Ccl4*, *f4/80*, *Il6, Tnf and p22phox* fold change relative to chow diet fed young female. **l** Heatmap of hepatic mRNA levels of *Ccl2, Ccl4*, *f4/80*, *Il6, Tnf and p22phox*. A representative of 2 individual experiments. Data represent means + SE. **b**, **c**, **e–i**, **k–m**. One-way ANOVA *P < 0.05, **P < 0.005 and ***P < 0.0005.

**Fig. 3:**
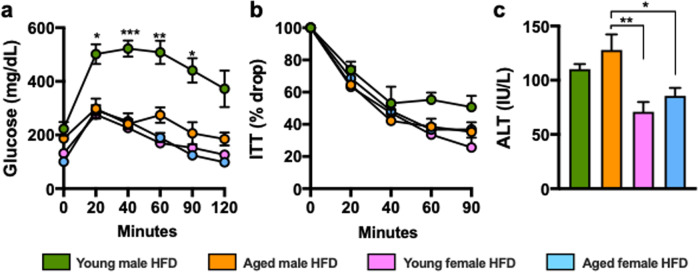
Gender impacts aging-driven effect on metabolic derangement severity. Comparison of data depicted in Figs. 1 and 2. **a** Glucose tolerance test (GTT). **b** Insulin tolerance test (ITT). **c** Serum Alanine transaminase (ALT) levels.

Gender differentially impacts pathophysiology of metabolic diseases^31–34^. Thus, we aimed to examine how aging impacts development of obesity and metabolic derangements in female mice (Fig. 2a). HFD fed aged, compared to young, female mice developed severe obesity (Fig. 2b). Aging coupled to HFD feeding in female mice promoted differential WAT distribution (Fig. 2c) which correlated with increased adipocyte size (Fig. 2d). Despite the dichotomous effects on weight gain aged and young female mice exhibited similar glucose handling and tolerance (Fig. 2e, f), fasting insulin levels (Fig. 2g) and mRNA expression of inflammatory mediators (e.g., *Ccl4, Ccl2*, *f4/80, Il6, Tnf* and *p22phox*) in WAT (Fig. 2h). Congruent with robust weight gain, HFD fed aged female mice exhibited increased hepatic triglycerides accumulation (Fig. 2i, j). However, these physiological changes in the liver were not sufficient to significantly alter liver function (Fig. 2k) or robustly promote liver inflammation (Fig. 2l). Notably, despite the induction of metabolic derangements in HFD fed aged female mice, the disease severity including glucose handling (Fig. 3a, b) and hepatocellular damage (Fig. 3c) was significantly reduced compared to HFD fed male mice. Together, these data indicate that despite gender differences in the severity of metabolic derangements under T_S_ housing conditions, aging-dependent changes may dominantly impact both gender- and diet-dependent effects.

### Thermoneutral housing enabled induction of severe obesity in female mice uncovers the impact of aging on metabolic disease severity

**Fig. 4:**
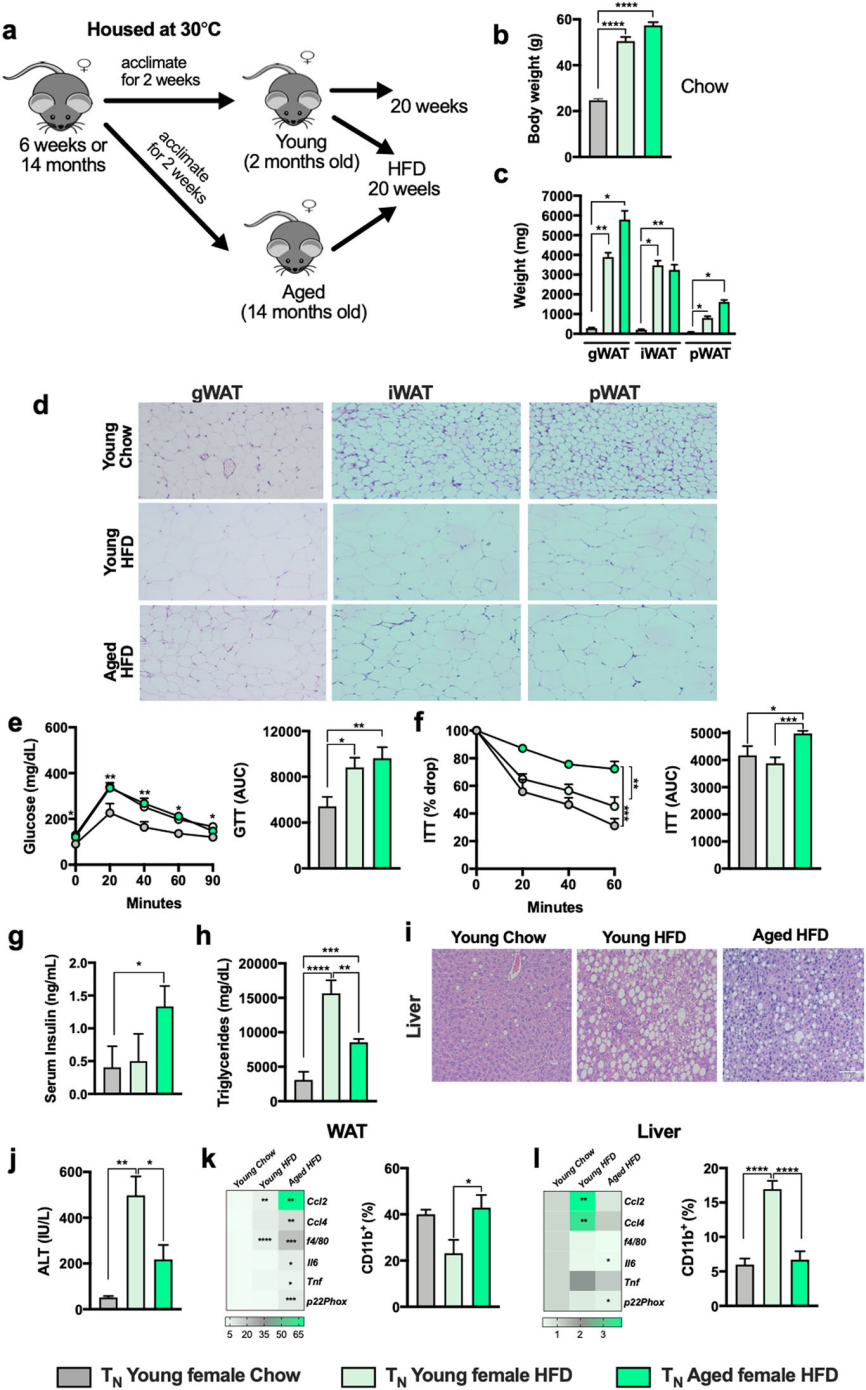
Thermoneutral housing uncovers the impact of aging on metabolic disease severity independent of weight difference in female mice. 2-month-old (Young) and 14-month-old (Aged) female C57BL/6 mice, (*n* = 4–9/condition) were housed at either 22 °C (T_S_) or 30 °C (T_N_) and fed Chow or HFD for up to 20 weeks. **a** Schematic representation of DIO model used. **b** Body weight at time of harvest. **c** White adipose tissue (WAT) weight: gonadal WAT (gWAT), inguinal WAT (iWAT) and perirenal WAT (pWAT). **d** gWAT, iWAT and pWAT hematoxylin eosin (H&E) staining. **e** Left, glucose tolerance test (GTT) and Right, GTT area under the curve (AUC). **f** Left, insulin tolerance test (ITT) and Right, ITT area under the curve (AUC). **g** Serum insulin levels. **h** Right-Heatmap of WAT mRNA levels of *Ccl2, Ccl4*, *f4/80*, *Il6, Tnf and p22phox* fold change relative to T_N_ chow diet fed young female; Left-Flow cytometry quantification of WAT infiltrating CD11b^+^ cells (CD45^+^). **i** Liver triglycerides. **j** Liver H&E staining. **k** Serum Alanine transaminase (ALT) levels. **l** Right-Heatmap of hepatic mRNA levels of *Ccl2, Ccl4*, *f4/80*, *Il6, Tnf and p22phox* fold change relative to T_N_ chow diet fed young female; Left-Flow cytometry quantification of hepatic infiltrating CD11b+ cells (CD45^+^). A representative of 2 individual experiments. Data represent means + SE. **b**, **c**, **e–i**, **k–m**). One-way ANOVA *P < 0.05, **P < 0.005, ***P  0.0005 and ****P < 0.00001.

Uncovering the impact of obesity and obesity-associated metabolic derangements in young female mice is hampered by the inability of HFD to promote severe obesity in routinely used experimental settings^11–13^. Use of thermoneutral housing (T_N_; 30 °C) allows for induction of severe obesity and NAFLD in female mice^13^. Thus, via utility of T_N_ housing, we aimed to examine how aging impacts development of obesity and metabolic disease severity in female mice. HFD-fed young (2 months-7 months at time of analysis) and aged (14 months-19 months at time of analysis) female mice housed at T_N_ (Fig. 4a) gained similar amount of weight and developed robust obesity (Fig. 4b). However, young and aged female mice housed at T_N_ conditions exhibited differential WAT distribution (Fig. 4c) which correlated with increased adipocyte size in aged mice (Fig. 4d). Both young and aged female mice housed at T_N_ developed mild glucose dysmetabolism (Fig. 3e, f), with higher insulin levels seen only in aged mice (Fig. 4g). In addition, T_N_ housed HFD-fed aged female mice had decreased hepatic steatosis (Fig. 4h, i) and hepatocellular damage (Fig. 4j). The dysregulated glucose handling and hepatic phenotype in aged female mice correlated with divergent inflammatory responses in gWAT (increased expression of *Ccl4, Ccl2*, *f4/80, Il6, Tnf* and *p22phox* and macrophage accrual in the gWAT; Fig. 4k) and liver (decreased expression of *Ccl4, Ccl2*, *f4/80, Il6, Tnf* and *p22phox* and macrophage accrual; Fig. 4l). These data suggest that T_N_ housing enabled modeling of severe obesity and metabolic dysfunction in young female mice (Table 1) and allowed for uncovering of aging effect on obesity and obesity-associated metabolic disease severity in female mice. Further, these data indicate that despite induction of severe obesity in young female mice at T_N_, the overall metabolic dysfunction in both young and aged female mice is milder compared to young HFD fed male mice.

**Table 1:** Thermoneutral housing, compared to standard housing, promotes severe obesity, metabolic dysfunction and amplifies tissue inflammation in young female mice.

	T_N_ vs T_S_ young females fed HFD
Obesity	Increased
Adiposity	Increased
Glucose Dysmetabolism	Increased
Steatosis	Increased
Hepatocellular Damage	Increased
Adipose Tissue Inflammation	Increased
Liver Inflammation	Increased

### T_reg_ and Tr1-like cell accumulation, and IL-10 production in aged mice is associated with reduced metabolic disease severity

**Fig. 5:**
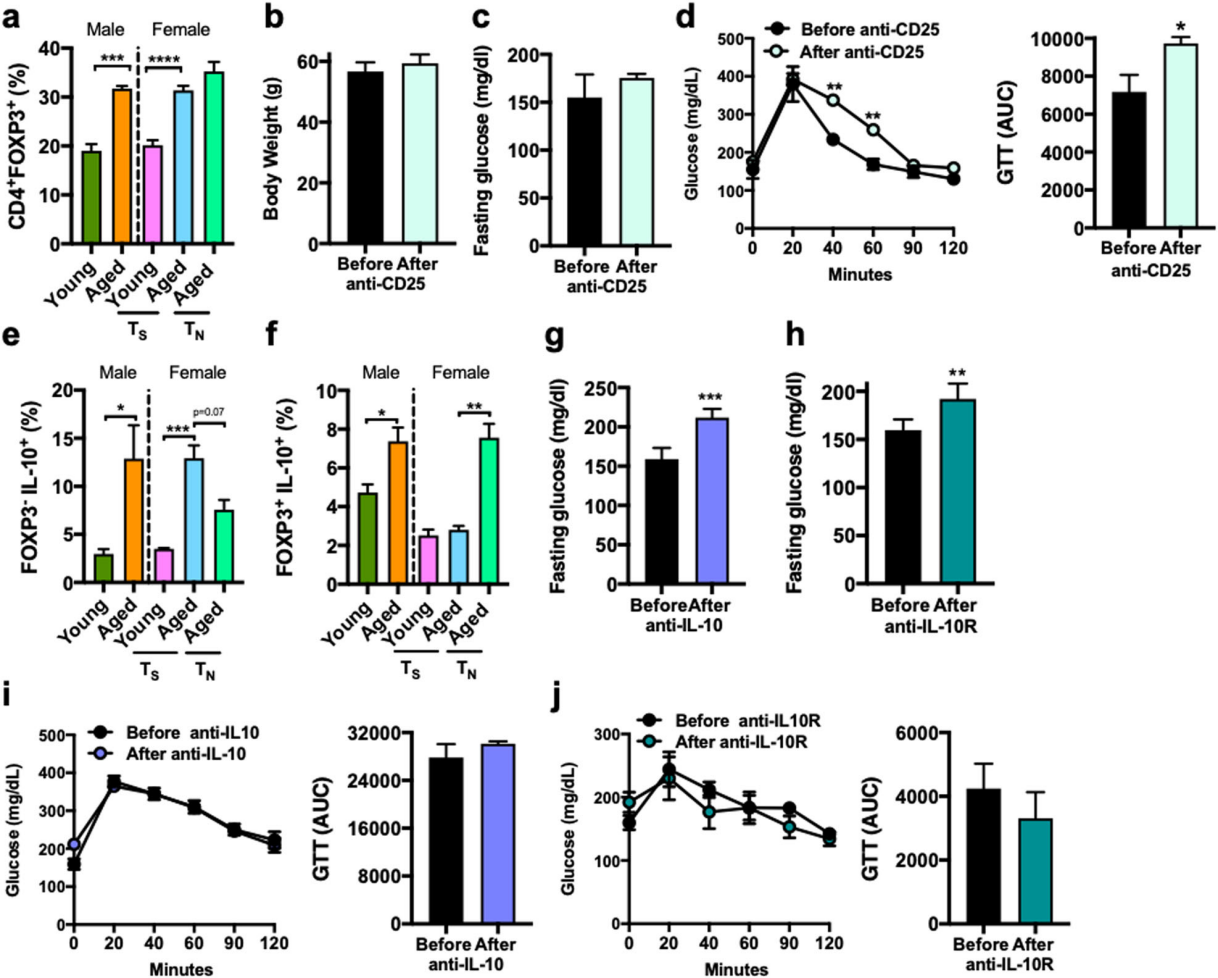
Aging promotes accumulation of T_reg_, Tr1-like cells and IL-10 production that may contribute to aging-associated mitigation of metabolic derangement severity. **a** 2-month-old (Young) and 14-month-old (Aged) male and female C57BL/6 mice, (*n* = 4–5/condition) were housed at 22 °C (T_S_) or 30 °C (T_N_) and fed Chow or HFD for up to 20 weeks. Flow cytometry quantification of splenic T_reg_ (CD3^+^CD4^+^FOXP3^+^). **b**–**d** Aged male mice (*n* = 3–4/group) were housed at 22 °C (T_S_) and fed HFD for 20 weeks. **b** Body weight at time of harvest. **c** At week 18, mice were treated, twice weekly, for 2 weeks, with anti-CD25 (100 μg/mice) neutralizing Ab. Fasting glucose levels before and after treatment. **d** Left, glucose tolerance test (GTT) and Right, GTT area under the curve (AUC) before and after anti-CD25 treatment. **e**, **f** 2-month-old (Young) and 14-month-old (Aged) male and female C57BL/6 mice, (*n* = 4–5/condition) were housed at 22 °C (T_S_) or 30 °C (T_N_) and fed Chow or HFD for up to 20 weeks. **e** Flow cytometry quantification of splenic Tr1-like cells (CD3^+^CD4^+^FOXP3^-^IL-10^+^) post PMA/Ionomycin (50 ng/ml and 1 mg/ml respectively). **f** Flow cytometry quantification of splenic IL-10 producing T_reg_ (CD3^+^CD4^+^FOXP3^+^) post PMA/Ionomycin (50 ng/ml and 1 mg/ml respectively). **g**–**j** Aged mice (*n* = 4–7/group) were housed at 22 °C (T_S_) and fed HFD for 20 weeks. At week 18, mice were treated, twice weekly, for 2 weeks, with anti-IL-10 (100 μg/mice) neutralizing Ab or with anti-IL-10R (500 μg/mice) neutralizing Ab. **g** Fasting glucose levels before and after anti-IL-10 treatment. **h** Fasting glucose levels before and after anti-IL-10R treatment. **i** Left, glucose tolerance test (GTT) and Right, GTT area under the curve (AUC) before and after anti-IL-10 treatment. **j** Left, glucose tolerance test (GTT) and Right, GTT area under the curve (AUC) before and after anti-IL-10R treatment. A single experiment. Data represent means + SE. **a, d**, **e** One-way ANOVA *P < 0.05, **P < 0.005 and ***P < 0.0005. **b**, **c**, **f–i** Unpaired Student *t* test *P < 0.05, **P < 0.005 and ***P < 0.0005.

Immune hypo-responsiveness in aging is associated with increased peripheral expansion of T_reg_ cells^17,35^. In obesity, T_reg_, via modulation of inflammation, improve insulin sensitivity, lower blood glucose, and reduce end-organ sequelae^36–38^. We hypothesized that increased T_reg_ frequency in aging limits obesity-associated metabolic disease severity. Both HFD fed aged male and female mice at T_S_ and T_N_, compared to young counterparts, exhibited increased frequency of splenic T_reg_ (defined as TCRβ^+^CD4^+^FOXP3^+^; Supplementary Fig. 2a) cells (Fig. 5a). To determine T_reg_ contribution to severity of metabolic derangements, we used anti-CD25 to deplete T_reg_ in vivo in aged HFD mice. Treatment with anti-CD25 did not alter HFD-driven body weight (Fig. 5b) or basal glucose metabolism (Fig. 5c) but was sufficient to impair exogenous glucose handling (Fig. 5d). Given the partial efficacy and modest effect of T_reg_ depletion (Supplementary Fig. 2b) on metabolic derangement severity in aged mice, we next hypothesized that additional cellular players may contribute to development of metabolic disease. Tr1-like cells are key producers of IL-10 and IL-10 production is increased in aging^19,39,40^. Notably, in our setting, aging was associated with increased IL-10 circulating levels particularly in female mice housed at T_S_ condition (Supplementary Fig. 2c). Increased splenic frequency of Tr1-like (defined as TCRβ^+^CD4^+^FOXP3^−^IL-10^+^; Supplementary Fig. 2d) cells was observed in both HFD fed male and female aged mice (Fig. 5e). However, the frequency of IL-10 producing T_reg_ cells (defined as TCRβ^+^CD4^+^FOXP3^+^IL-10^+^; Supplementary Fig. 2d) was only increased in HFD fed male and T_N_ housed female aged mice (Fig. 5f). Lack of reagents specific for depletion of Tr1-like cells led us to examine the impact of IL-10 signaling neutralization on metabolic disease severity in aging. Antibody-mediated neutralization of either IL-10 or IL-10R in vivo in aged mice did not alter HFD-driven body weight (Supplementary Fig. 2e, f). Importantly, blockade of IL-10 or IL-10R exacerbated basal glucose metabolism (Fig. 5g, h) without impacting exogenous glucose handling (Fig. 5i, j). Collectively, this data suggests that independent of gender, aging drives peripheral expansion of immunoregulatory networks including T_reg_ and Tr1-like cells and IL-10 that could actively and cumulatively participate in limiting the severity of obesity-associated metabolic derangements.

## Discussion

The unabated prevalence of obesity in all age groups, including older adults (>65 years of age), is directly associated with increased body fat and adipose distribution^41^ and higher rates of T2D and NAFLD^42^. Published evidence suggest that gender differentially impacts pathophysiology of metabolic derangements^5^. Thus, understanding the impact of gender in metabolic disease development and severity is of immense clinical importance. In the present study, using T_S_ and T_N_ housing and obesogenic diet feeding, for the very first time we compared the impact of aging and gender on metabolic disease severity independent of weight difference. We show that aging in male and female mice altered body weight and augmented adipose tissue mass under homeostatic conditions. This is in agreement with data reporting that ad libitum fed male and female mice experience a peak in body weight around 20–24 months of age and display fat mass expansion^43–45^. Body weight in mice remains relatively stable up until the end of life, however, mice display decline in body weight and fat composition during advanced age (26 months and up) associated with health decline and proximate death^46^.

Our data demonstrates that aging in male mice promoted altered adipose distribution without increasing body weight gain or promoting glucose dysmetabolism under obesogenic diet feeding regimen. This was a rather unexpected finding, as chow diet fed male mice exhibited mild AT inflammation (e.g., TNF) which could predispose towards worse metabolic outcomes. However, both detrimental and protective effects of TNF in obesity-associated metabolic disease and adipose tissue homeostasis are reported. The impact of TNF on adipose tissue function in aging however remains unknown. Notably, TNF is important for healthy adipose tissue expansion and remodeling^47^ as well as the initiation and progression of obesity-driven insulin resistance^15,48^. Obese mice lacking either TNF or its receptor are protected from insulin resistance^49^. Additionally, TNF promotes lipolysis and release of free fatty acids by adipocytes, which in turn increases hepatic gluconeogenesis^50^. Thus, modestly increased TNF levels in WAT of middle-aged/aged male mice could contribute to adipose tissue expansion in aging, marginal glucose dysmetabolism, and increased hepatic triglycerides accumulation.

The paucity of glucose dysmetabolism in aged obesogenic diet fed male mice suggests that the aging environment may be a dominant factor in regulating the severity of metabolic derangements. Similarly, aged male mice fed obesogenic diet had decreased hepatic steatosis, yet similar hepatocellular damage compared to young male counterparts. The dichotomous effects in the liver could be attributed to increased sensitivity of aged hepatocytes to secondary stimuli (e.g., lipid accumulation, ROS and inflammatory mediators). Aging is associated with decreased liver mass^51^ and increased oxidative stress and ROS accumulation^52^, which leads to the collapse of the hepatic mitochondria and apoptosis. Combined, these effects may explain the similar ALT levels between young and aged obese mice despite reduced hepatic steatosis. Future studies aimed at the mechanistic interrogations of these pathways are needed. The severity of obesity-associated metabolic diseases in aging is somewhat controversial with both reduced^53–57^ and exacerbated disease severity^58,59^ reported. Lean aged mice displayed reduced insulin sensitivity compared to young lean mice— something trending in our studies. Notably, in context of HFD feeding our data are in agreement with the published reports describing reduced metabolic disease severity in aging^53–57^. The potential rationale for divergent results could be attributed to limited weight gain by young male mice on a HFD^58^ or by the differences of the obesogenic diet used^59^.

Previous studies examining the impact of aging on obesity-driven metabolic disease severity have primarily utilized male mice^53–55,58^. Reports focused on the impact of aging on metabolic disease severity in female mice have been limited by commonly observed protection from obesogenic diet-driven weight gain in this setting. Specifically, young female mice exhibit a very modest weight gain when fed obesogenic diet. This is directly coupled to limited metabolic alterations including minor WAT and liver tissue inflammation, improved insulin sensitivity, and lower lipid deposition compared to male counterparts^13,60,61^. Our data supports these findings and demonstrate that young female mice fed obesogenic diet do not develop severe obesity (weighing less than 35 g) while aged female mice became severely obese (weighing above 45 g) even at T_S_ housing. Despite severe obesity aged female mice exhibited normal insulin responsiveness, and mild glucose dysmetabolism and hepatocellular damage. Importantly, such effects resembled those observed in obese aged male mice and were significantly milder in severity compared to obese young male mice. Together, these findings suggest that increase in body weight in aged individuals might be counterbalanced by aging effects which dominantly regulate severity of metabolic derangements. This is in agreement with clinical findings invoking that overweight older adults (BMI above 25) live longer than slender older counterparts^62^. Thus, further definition of underlying mechanisms in these settings warrants full attention.

Lack of efficient diet-induced obesity in female mice is in part dependent on housing temperature conditions. Traditionally, research mice are housed at 22 °C, a temperature below their thermoneutral zone^14^. Housing under such conditions promotes thermal stress requiring increased energy expenditure for adaptive thermogenesis^63–66^ that may ultimately obscure the outcomes of weight gain and metabolic derangement studies^67–70^. Importantly, T_N_ housing allowed for the development of severe obesity in both young and aged female mice, which could be a result of diminished activation of thermogenic pathways during T_N_ housing^71,72^. Given that such effects are dominant in female mice, T_N_ housing might provide a model for a better understanding of the impact of gender on thermogenic activation. Importantly, housing mice at thermoneutrality (30 °C) relieved cold stress-associated changes in physiologic, metabolic and immune functions^73,74^ which promotes obesity and NAFLD severity^13^. However, despite severe obesity, our data show that aged female mice exhibited similar glucose dysmetabolism and decreased hepatocellular damage compared to their young counterpart and such effects correlated with the inflammatory profile in WAT and liver. Our studies utilized the power of T_N_ housing experimental model to examine the impact of aging on weight gain, development of metabolic derangements, and altered immune responsiveness in obese young and middle-aged/aged female mice. Although, we did not directly compare the effect of aging between male and female mice housed at T_N_ condition we would expect that aging, even under T_N_ housing, would similarly play a beneficial role in HFD-driven metabolic outcomes in males mice. Together these data would suggest that the aging limits obesity-associated metabolic disease outcomes regardless of the environmental conditions.

Gender influences development of obesity and metabolic diseases. Our data shows that aged obese female mice housed either at T_S_ or T_N_, compared to their aged male counterparts, gained more weight and had increased adiposity but exhibited attenuated diet-induced glucose dysmetabolism and hepatocellular damage. Previous studies have shown that aged female rats, compared to male counterparts, are protected from age-related insulin resistance, hepatic lipid deposition, and WAT inflammation—something associated with greater capacity of females to undergo WAT expansion and their ability to maintain adiponectin levels and preserve leptin sensitivity with aging^75^. Additionally, compared to male mice, aged female mice, had better insulin sensitivity and reduced steatosis after obesogenic diet feeding^60^. Notably, this is in line with human studies that show differential effects of gender on metabolic derangements through lifespan. Specifically, the prevalence of obesity and metabolic diseases are higher among teenage males than females^6^, while in adulthood females exhibit increased adiposity compared to males. Additionally, WAT distribution varies between males and females, with males gaining adipose in visceral depots compared to pre-menopausal women that gain adipose in subcutaneous depots, which is lost after menopause^7^. Importantly, other studies suggest that post-menopausal women have higher prevalence of obesity-associated metabolic disorders^8^.

The underlying mechanisms whereby females may have similar or greater adiposity and exhibit lower propensity than males to age-related metabolic alterations are not known, beyond the plausible implication of sex hormones. Estrogen removal in animals or menopause in women is associated with some metabolic disturbances including hepatic triglycerides accumulation or an increase in HOMA-IR index^76–78^. Estrogen protects against the development of obesity, and whole body estrogen receptor deletion leads to accumulation of visceral AT and development of metabolic syndrome^79,80^. Lack of estrogen receptor only in adipocytes led to fat expansion but reduced inflammation^79^, which correlates with the notion that estrogen influence the effector functions of immune cells known to promote metabolic derangements (e.g., macrophage, NK and T cells)^81^. Estrogen also regulates expression of enzymes (e.g., lipoprotein lipase, hormone-sensitive lipase) that regulate lipid metabolism in adipose and liver tissues^82^. Thus, estrogen levels in aged female mice, regardless of housing conditions, may contribute to the severity of metabolic derangements in our models. Although we did not examine whether female mice employed in our studies had gone through menopause, reduced concentrations of estradiol (E2) during the estrous cycle, no detectable midday preovulatory elevation of E2, and an attenuated preovulatory increase of progesterone has been reported in middle-aged female C57BL/6 mice compared to young mice^83^. These data suggest that the female mice employed in our studies (19 months of age) have likely initiated hormonal changes associated with menopause which may influence the development of obesity and metabolic disease.

Aging is characterized by a persistent low-grade immune activation that is implicated in detrimental processes including metabolic diseases^84^. In addition, aged mice display exacerbated systemic inflammation^85^ and WAT inflammation^86^. Inflammation is strongly implicated in insulin resistance^87^. Our findings show that aging in chow fed mice is associated with increased expression levels of CCL2 (macrophage recruiting chemokine), p22phox (an indicator of oxidative stress) in the WAT. However, despite increased baseline inflammation aged male and female mice fed obesogenic diet had decreased WAT inflammation and exhibited protection from glucose dysmetabolism. Given that inflammatory mediators associated with metabolic diseases could be regulated by the aging environment and that aging promotes peripheral expansion of multiple types of immunoregulatory cells and mediators including T_reg_^35^ and Tr1 cells^19^ it is plausible that such immune permutations impact metabolic disease severity.

Our data show that aged, obese male and female mice exhibit expansion of peripheral T_reg_ cells. Aging is associated with a gradual increased in proportion of T_reg_ in WAT^36,88^, followed by a precipitously decreased T_reg_ frequency in advanced aged mice^88^. Importantly, T_reg_ accrual in WAT in male mice was reduced during obesogenic diet feeding^36^. In contrast, obesogenic diet feeding promoted WAT T_reg_ expansion in T_S_ housed young female mice^89^, which may explain the limited induction of metabolic diseases. While we did not directly examine WAT T_reg_ cells in our model_,_ expansion of T_reg_ cell numbers in WAT at homeostasis has been demonstrated^20^. Despite these key observations, the contribution of peripheral expansion of T_reg_ cells in obesity and aging remains underdefined. As T_reg_ cells play a protective role in insulin sensitivity and energy homeostasis in obesity^38^, Increased adipose inflammation was observed in T_reg_-depleted mice, whereas glucose metabolism was ameliorated in obese mice after adoptive transfer of T_reg_ cells^36,37^.

Obesity is associated with increased IL-6 levels^90^ and IL-6 can drive T_reg_ expansion in aging^91^. Thus, it is possible that increased IL-6 production in obesity and aging additively contributes to peripheral T_reg_ accrual in both male and female mice. Although significant, the effect on glucose metabolism upon depletion of T_reg_ using anti-CD25 was modest. This may in part be explained by the fact that a proportion of T_regs_ in aged, relative to young, mice have decreased expression of CD25^92,93^, yet this CD25^low^ T_reg_ population in aging retains comparable functional properties to the traditional CD25^+^ T_regs_^35^, which was reflected in the lack of full peripheral T_reg_ depletion in our studies. Thus, further, in depth definition of T_reg_ phenotypes, characteristics and function in aging is needed. In addition to T_reg_, IL-6 also induces the generation of IL-10-producing Tr1 cells that are known to suppress autoimmune tissue inflammation^94^. Aging is characterized by increased systemic levels of IL-10^95^. Increased IL-10 production is associated with healthy aging and insulin-sensitizing effects^21^. However, in our study, IL-10 neutralization had minor effects on glucose metabolism. These findings highlight the broader involvement of immune system hyporesponsiveness, something beyond the effects of IL-10, T_reg_ and Tr1-like cells alone, in modulating severity of metabolic derangements in aging and obesity.

In conclusion, our findings show that aging dominantly impacts both gender- and diet-dependent pathways that promote the severity of metabolic derangements. The age-dependent changes in the immunoregulatory mechanism could explain the aging-driven protection, pinpointing the mechanisms of attenuation of metabolic diseases. Future exploitation of such mechanisms holds great implications for metabolic disease research and development of novel therapeutic strategies.

## Supplementary information

SUPPLEMENTARY FIGURE LEGENDS

Supplementary Fig. 1

Supplementary Fig. 2.
