# Supplementary material for: Aging mitigates the severity of obesity-associated metabolic sequelae in a gender independent manner
Source: Nutr Diabetes. 2021 Jun 7;11:15. doi: 10.1038/s41387-021-00157-0 (PMC8184786; doi:10.1038/s41387-021-00157-0)
Supplement: Supplementary file 1 — SUPPLEMENTARY FIGURE LEGENDS [file 41387_2021_157_MOESM1_ESM.docx]

**SUPPLEMENTARY FIG. LEGENDS**

**Supplementary Fig. 1. Aging mitigates metabolic disease severity in male mice.**

Extended data associated with Figure 1. (**a**) Body weight gain. (**b**) Weekly food intake. (**c**) Serum insulin levels at time of harvest. (**d**) Heatmap of WAT mRNA levels of *Ccl2, Ccl4*, *f4/80*, *Il6, Tnf and* *p22phox* fold change relative to HFD fed young male (**e**) Comparison of the ratio of WAT mRNA levels of *Ccl2, Ccl4*, *f4/80*, *Il6, Tnf and* *p22phox* between Chow vs HFD in Young and Aged male mice. (**f**) Heatmap of hepatic mRNA levels of *Ccl2, Ccl4*, *f4/80*, *Il6, Tnf and* *p22phox* fold change relative to HFD fed young male. Data represent means + SE. (A-E) Unpaired Student *t* test *P<0.05 and ***P<0.0005.

**Supplementary Fig. 2. Aging promotes accumulation of T_reg_, Tr1-like cells and IL-10 production that may contribute to aging-associated mitigation of metabolic derangement severity.**

Extended data associated with Figure 5. (**a**) A representative dot plot of splenic T_reg_ quantification by Flow cytometry. (**b**) A representative dot plot of splenic T_reg_ cells (FOXP3^+^) after anti-CD25 treatment. (**c**) Serum IL-10 at time of harvest. (**d**) A representative dot plot of splenic T_reg_ IL-10^+^ (FOXP3^+^IL-10^+^) and Tr1-like cells (FOXP3^-^IL-10^+^) quantification by Flow cytometry post PMA/Ionomycin (50 ng/ml and 1 mg/ml respectively). (**e**) Body weight before and after anti-IL-10R treatment. (**f**) Body weight before and after anti-IL-10 treatment. Data represent means + SE. (**a**) One Way ANOVA *P<0.05.
