# Supplementary figures and images for: Aging mitigates the severity of obesity-associated metabolic sequelae in a gender independent manner
Source: Nutr Diabetes. 2021 Jun 7;11:15. doi: 10.1038/s41387-021-00157-0 (PMC8184786; doi:10.1038/s41387-021-00157-0)

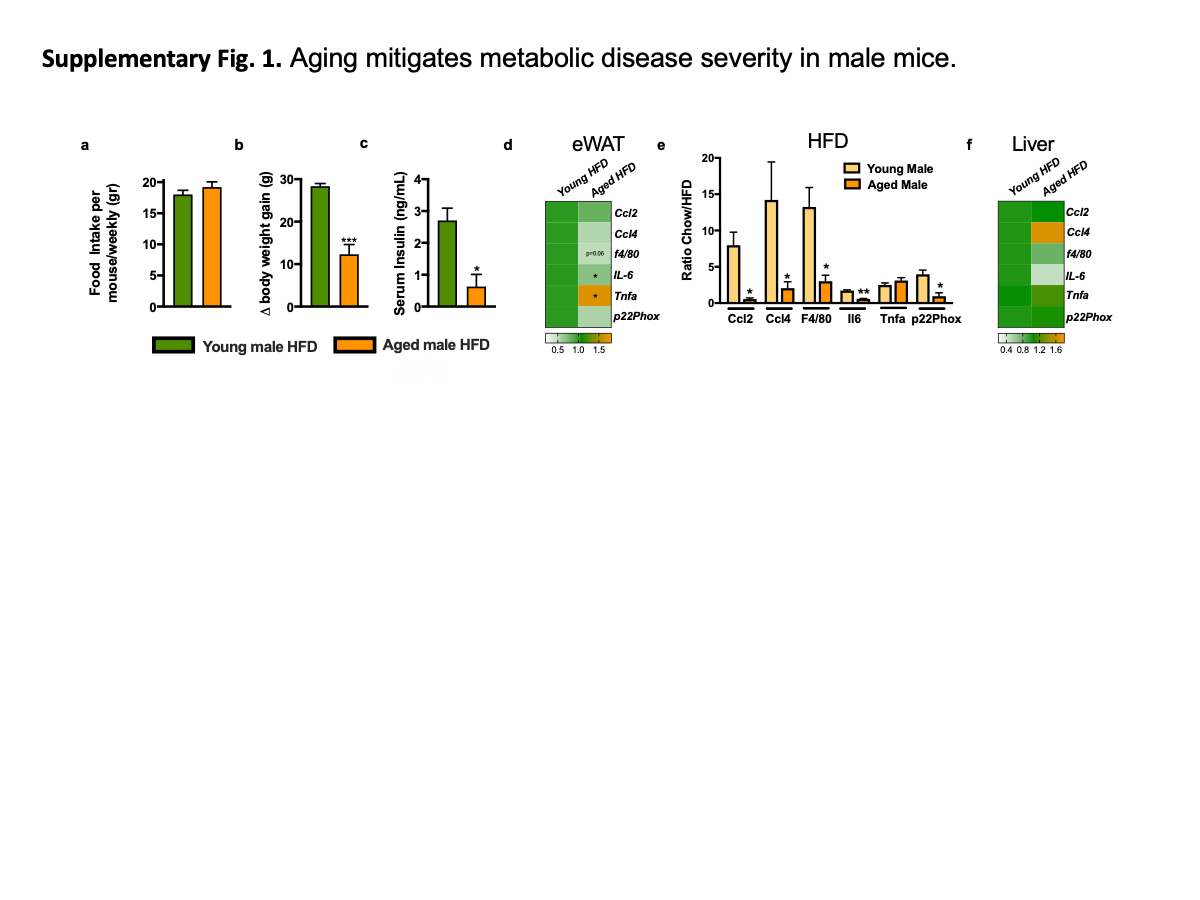

Supplement: Supplementary file 2 — Supplementary Fig. 1 [file 41387_2021_157_MOESM2_ESM.tif]

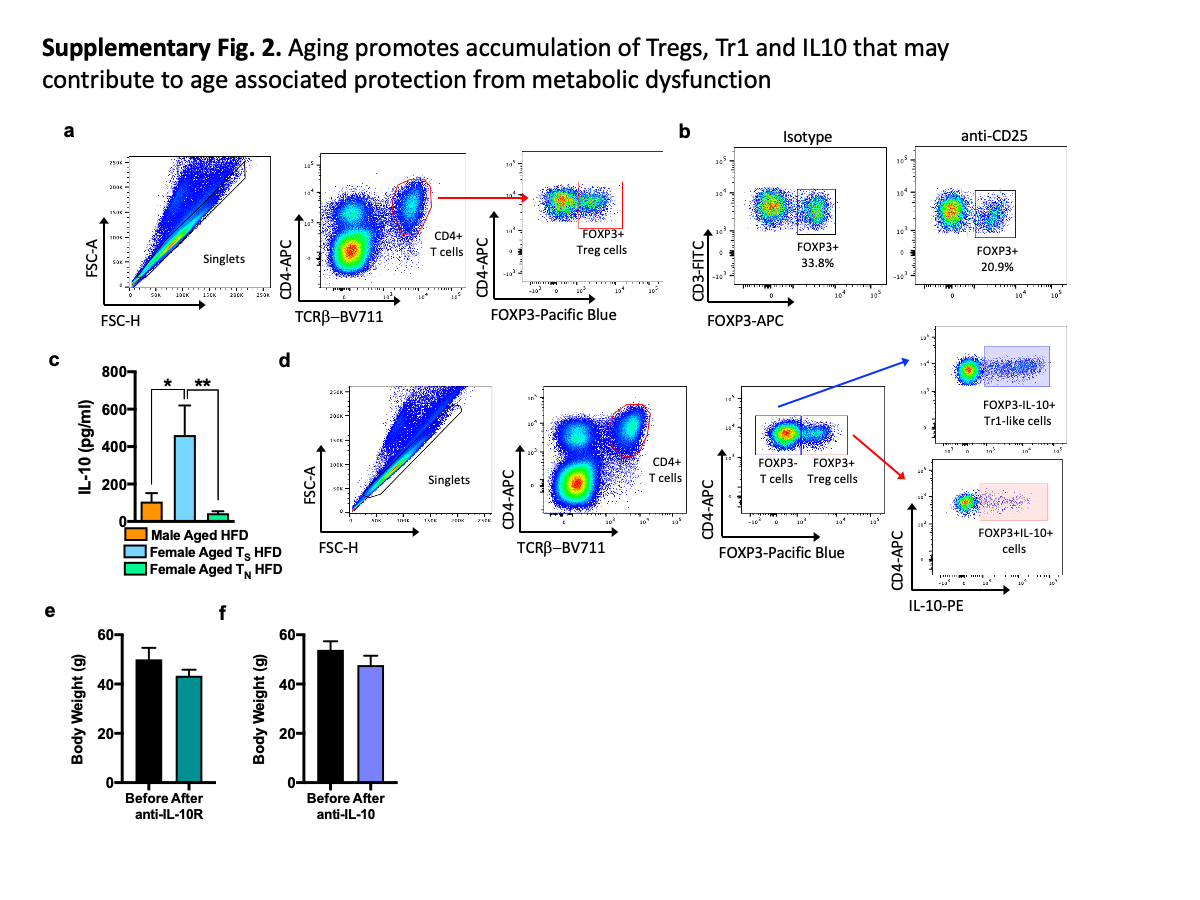

Supplement: Supplementary file 3 — Supplementary Fig. 2. [file 41387_2021_157_MOESM3_ESM.tif]
